# A Pilot Randomised Clinical Trial Comparing a Short-Term Perioperative Prophylaxis Regimen to a Long-Term Standard Protocol in Equine Colic Surgery

**DOI:** 10.3390/antibiotics10050587

**Published:** 2021-05-16

**Authors:** Sabita Diana Stöckle, Dania A. Kannapin, Anne M. L. Kauter, Antina Lübke-Becker, Birgit Walther, Roswitha Merle, Heidrun Gehlen

**Affiliations:** 1 Equine Clinic: Surgery and Radiology, Freie Universität Berlin, 14163 Berlin, Germany; dania.kannapin@t-online.de (D.A.K.); heidrun.gehlen@fu-berlin.de (H.G.); 2 Robert Koch Institute, Advanced Light and Electron Microscopy, 13353 Berlin, Germany; a.kauter@online.de (A.M.L.K.); waltherb@rki.de (B.W.); 3 Institute of Microbiology and Epizootics, Freie Universität Berlin, 14163 Berlin, Germany; antina.luebke-becker@fu-berlin.de; 4 Institute for Veterinary Epidemiology and Biostatistics, Freie Universität Berlin, 14163 Berlin, Germany; Roswitha.Merle@fu-berlin.de

**Keywords:** colic, laparotomy, perioperative antibiotics, surgical site infection, colitis, haemolytic anaemia, horse

## Abstract

Background: For surgical interventions classified as clean or clean-contaminated, including laparotomy, guidelines in human and veterinary medicine recommend a short-term perioperative antibiotic prophylaxis (PAP). In equine colic surgery, however, PAP commonly exceeds 24 h. Objectives: The aim of this study was to compare a single-shot to a 5-day lasting PAP considering surgical site infections (SSI) and other adverse effects probably associated with the particular antimicrobial regimen. Study design: The study was designed as a randomised non-inferiority pilot study including horses subjected to colic surgery while receiving one of two distinct PAP regimens. Methods: All horses (*n* = 67) included in the study received the standard physical examination before and after surgery. Colic surgery was performed according to the current standard of the clinic. Horses were randomly assigned to two groups, receiving either the “single-shot” or the “5-day lasting” antibiotic prophylaxis. The “single-shot” group (*n* = 30) received penicillin and gentamicin only once before and, if needed, during surgery, whereas the “5-day lasting” group (*n* = 37) received antibiotics for five days. In addition to the standard laboratory examinations, serum amyloid A and fibrinogen were determined preoperatively and during five days after surgery. SSI, postoperative colitis and haemolytic anaemia were classified as postoperative complications potentially related to antibiotic use. Results: The outcome of this preliminary non-inferiority clinical trial showed that the occurrence of postoperative adverse events (i.e., SSI, postoperative colitis and haemolytic anaemia) lacked significant differences between the study groups. Main limitations: The main limitations of this study are the limited group sizes and our inability to blind the study. Conclusions: Single-shot PAP seems to be an alternative approach considering the 5-day lasting protocol commonly used in equine abdominal surgery. However, a proper hygiene management together with a close clinical and laboratory monitoring of the equine patient is indispensable.

## 1. Introduction

The abdominal pain syndrome complex caused by disorders of the gastrointestinal tract in horses is commonly referred to as “colic” [1]. An annual incidence of 4.2% was reported for the prevalence of equine colic in the United States, including 1.4% of horses which required emergency laparotomy [1]. According to Cruse and Foord [2], abdominal surgery, such as equine exploratory laparotomy, is either classified as a clean (i.e., without enterotomy and/or resection) or clean-contaminated (i.e., with enterotomy and/or resection) surgical procedure. Surgical site infections (SSI) are among the most common adverse events after colic surgery, alongside postoperative colic/pain, postoperative ileus, severe endotoxaemic shock, jugular thrombophlebitis and postoperative colitis [3,4,5,6].

Incisional wound healing is not only influenced by the preoperative preparation of the surgical site, skills of the surgeon, the procedures and techniques used during the intervention [7,8], but also by the systemic health state of the horse. Since the horse’s recovery and return to athletic function can be impaired by complications in incisional healing such as SSI [7,8], prevention of SSI and other adverse effects is crucial after colic surgery.

Furthermore, the rational use of antibiotics is considered a key factor to maintain the effectiveness of important antibiotic agents, which is of utmost importance, considering the limited antibiotics currently available for horses, at least in Germany [9,10,11,12]. Moreover, decreasing the inevitable selective effects of antibiotics, by enhancing their rational use, counteracts the spread of antimicrobial resistance among pathogens known to commonly cause SSI in horses. The latter includes Gram-positive and -negative bacteria residing on the skin and mucosal surfaces of horses [13]. Considering the onset of postoperative SSI, antibiotics are administered in the immediate perioperative period, a precaution which is known as perioperative antibiotic prophylaxis (PAP) [14,15].

A “single-shot” antibiotic application is considered sufficient in human medicine in cases of clean-contaminated surgery such as laparotomy [14]. However, in equine laparotomy, the most commonly applied perioperative antibiotic regimens seem to regularly exceed 24 h [16,17]. Equine patients have special needs concerning their environment and housing (i.e., large stables with non-skid floors, litter, dust and manure). Thus, keeping pathogenic bacteria able to cause postoperative infections at bay is often challenging compared to the situation associated with clinical environments of human or even small animal healthcare facilities [9,10]. These considerations may have an impact on the surgeons’ decision on prolongation of the actual PAP.

A single antibiotic dose seems sufficient to avoid SSI for various kinds of surgery for human patients lacking additional risk factors, such as advanced age, a chronic disease or pre-existing infections [15,18,19]. Nonetheless, if a surgical intervention exceeds 3 h or antibiotics which require frequent re-dosing (≥3x/day) are used, a repetition dose is recommended after two half-lives of the antibiotic agent selected [14]. Antibiotics which are administered beyond the timeframe of 24 h postoperatively are generally considered as therapy [14,15]. Different studies have already verified the lack of benefit of prolonged (>24 h) antibiotic administration in cases of clean-contaminated human surgeries [15,18,19].

A previous study found no significant difference between a 72 and 120 h antibiotic protocol regarding SSI rates after equine colic surgery [17]. So far, the rates reported for SSI after median laparotomy in horses range from 15% to 50% [6,17,20,21,22,23], with even increased rates reported for horses undergoing re-laparotomy (83%) [17,24].

Healthcare-associated infections in horses have often seemed to be associated with multidrug-resistant (MDR) bacteria [10,25,26], including SSI [25,27]. In addition, our previous studies have revealed that horses entering an equine clinic may already be colonised with MDR bacteria such as methicillin-resistant *Staphylococcus aureus* (MRSA) (i.e., nostrils, 3.5%) [27,28] and extended-spectrum beta-lactamase (ESBL)-producing Enterobacteriaceae (i.e., intestinal carriage, 10.7%) [26,29].

Therefore, it seems essential to promote the rational use of antibiotics and even reconsider standardised protocols for PAP in equine surgery.

The primary focus of this pilot randomised clinical trial with a non-inferiority design was to determine that a short-term PAP is not associated with more clinical adverse events (i.e., SSI, postoperative colitis and drug-induced haemolytic anaemia) than a conventional 5-day lasting PAP protocol in equine colic surgery (=non-inferiority trial). The secondary outcomes included assessment of blood parameters and bacteria associated with SSI in both study groups.

## 2. Material and Methods 

### 2.1. Ethical Statement

According to the German regulation authorities for research with animal subjects, the comparison of two antibiotic regimens does not require approval (Landesamt für Gesundheit und Soziales, Berlin, 18.04.2017). Written owner’s consent to involve their horses in the study was obtained during the admission process at the clinic.

### 2.2. Study Population

Non-inferiority trial calculation (Sealed Envelope Ltd. 2012. Power calculator for binary outcome non-inferiority trial. [Online, last accessed 03/2021] Available from: https://www.sealedenvelope.com/power/binary-noninferior/) was used to calculate the sample sizes for this pilot study. Considering the literature on SSI following colic surgery [6,17,20,21,22,23] a conservative minimum of 10% for SSI (and other adverse events) was assumed for both groups, resulting in the assumption of success (lack of SSI and other adverse events) in 90% of the equine patients. Based on the wide range of rates reported for the most frequently occurring adverse event following colic surgery SSI (15% to 50% [6,8,17,20,21,22]) and the pilot feasibility character of the study, the study power was set to 60% and the inferiority limit to 15%. With a 5% significance level, a sample size of 29 was calculated per group (total: 58). In order to strengthen the study power, we decided to include more equine patients, if possible. However, when benzylpenicillin for veterinary use was (temporarily) not available from September 2019 forward and the remainders ran out in 2020, the study was terminated.

Horses were randomised to the PAP regimens in a 1:1 ratio. Randomisation was performed in the theatre after wound closure to prevent performance bias using sealed envelopes (1:1 ratio) which have been stored in a closed box. The box content was checked with respect to coherence by counting the remaining envelopes each week. This was an open-label trial as the different antibiotic regimens prevented blinding.

Colic patients (*n* = 99) that were presented to the clinic and required laparotomy from January 2018 to February 2020 were considered as study participants. Metadata collected for each case included breed, age, gender and weight (Supplemental Table S1). The time of general anaesthesia, the medical indication for surgical intervention and, if applicable, the necessity for an enterotomy and/or intestinal resection was recorded for each patient. Surgeries in which an enterotomy and/or a resection was required were considered as clean-contaminated surgeries, whereas surgeries without enterotomy and/or resection were considered as clean surgeries. Since an intestinal resection is generally more invasive than an enterotomy, the two procedures were treated separately during the statistical evaluation. Horses (*n* = 11) which suffered from an infectious disease preoperatively or from any other condition requiring continuing antibiotic treatment were excluded from participation. Furthermore, all horses receiving antibiotics which were not in strict compliance with the study protocol (i.e., during nightshifts, due to intraoperative contamination) were removed from both study groups (*n* = 12). In addition, horses that were euthanised (during surgery or shortly afterwards), those subjected to a second surgical intervention within three days postoperatively, or horses that did not survive the third day after surgery (day of first bandage change, *n* = 9) were not considered for statistical evaluation. Otherwise, horses were further investigated regarding SSI, postoperative colitis and clinical signs of haemolytic anaemia. None of the horses were euthanised because of an incisional wound infection. 

Following up, we contacted equine patient owners over telephone (March 2020), asking whether there had been any signs of wound-healing disorders or other adverse events after the hospital discharge of their horses. A period of 3 weeks to 25 months had passed between the surgical intervention and these follow-up calls.

### 2.3. Clinical Examinations and Diagnostic Laparotomy

All horses were examined physically, transrectally and sonographically upon arrival. A nasogastric tube placement was performed on all horses with signs of acute colic as part of the standard hospital procedure. In addition to the common laboratory diagnostics (complete blood-count and blood-gas analysis), serum amyloid A (SAA) and fibrinogen values were determined to monitor increased inflammatory reaction, possibly indicating adverse events, including SSI.

Surgical intervention in horses with colic was performed according to the current in-house protocol. The muscle layer was closed in a continuous pattern with Polysorb loop (Lactomer 9-1) 2 USP. Each surgical site was flushed with 0.9% sterile sodium chloride (KOCHSALZLÖSUNG 0.9% B. Braun Spüllsg. Ecotainer, B. Braun Melsungen AG, Melsungen/Germany) before applying intra-cuticular sutures with Monocryl 2-0. The surgical wound was covered (NOBARAPID^®^, NOBAMED Paul Danz AG, Wetter (Ruhr)/Germany), fixed with Hypafix^®^ (BSN medical GmbH, Hamburg/Germany) and an abdominal bandage consisting of adhesive bandages (RUDALASTIK^®^, NOBAMED Paul Danz AG, Wetter (Ruhr)/Germany) was applied before the horse was transported to the recovery box. Each horse was closely monitored after surgery and further medical care was individually adjusted. Every patient received flunixin-meglumine (1.1 mg/kg, twice a day, (BID) Flunidol^®^ RPS, CP-Pharma Handelsgesellschaft mbH, Burgdorf/Germany) for 5 days after surgery. To prevent catheter-related thrombosis, horses received either low-molecular heparin (tinzaparin 50 IU/kg once daily (SID), INNOHEP multi 10.000 Anti-Xa IE/mL, 2 mL Dsfl, Kohlpharma GmbH, Merzig/Germany) or un-fractioned heparin (Heparin-Natrium Braun “Multi” 10,000 IE/mL, B. Braun Melsungen AG, Melsungen/Germany) in decreasing dosages, starting with 100 IU/kg BID. Due to the European veterinary medicine legislation, only horses which were excluded from human consumption were treated with doses of low-molecular heparin. Postoperative check-ups during the following 5 days included, beyond others, a complete blood count, evaluation of the acid-base status, determination of inflammatory markers (i.e., SAA and fibrinogen) and electrolyte ratios twice a day. A final regular check was accomplished on the tenth day.

**Figure 1:**
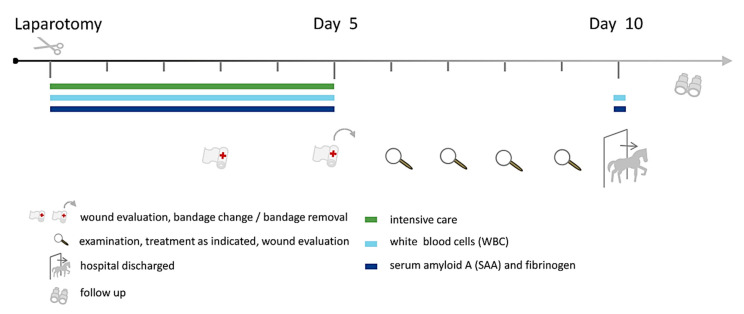
Illustration summarising the general post-surgical care and examinations applied to horses with colic surgery.

On the third day after surgery, the abdominal bandage was routinely changed. Additional changes were carried out whenever considered necessary. In most cases, the abdominal bandage was removed on day 5. A standardised wound evaluation was performed on days 3, 5 and 10 after surgery. An SSI was diagnosed by the veterinarian in charge, considering, beyond others, seropurulent or purulent exudation from the incisional site and/or suture dehiscence with or without a positive microbiological culture result. Complications other than SSI, which are possibly associated with the antibiotic protocol, were defined as postoperative colitis and drug-induced haemolytic anaemia. A comprehensive overview illustrating the post-surgical follow-ups is presented in Figure 1.

### 2.4. Perioperative Antibiotic Prophylaxis

The PAP in this study included sodium penicillin G (22,000 IU/kg, Penicillin-G-Natrium, bela pharm GmbH und Co. KG, Vechta/Germany or INFECTOCILLIN^®^ parenteral 10 Mega, INFECTOPHARM Arzneimittel und Consilium GmbH, Heppenheim/Germany) and gentamicin (6.6 mg/kg, Genta 100 mg/mL, CP-Pharma Handelsgesellschaft mbH, Burgdorf/Germany), as reported for colic surgery previously [17,30,31]. In each case, the antibiotics were administered under general anaesthesia 30–60 min before the initial incision. Since colic surgeries commonly last more than 3 h, sodium penicillin G was re-dosed after two of the agent’s half-lives (22,000 IU/kg).

While horses belonging to the SSG did not receive further antibiotics, 5DG horses received antibiotics for 120 h in total (penicillin (22,000 IE four times daily QID) and gentamicin (6.6 mg/kg SID)). The latter PAP regimen has been reported previously [17]. It was not possible to blind the study due to medical reasons. 

### 2.5. Statistical Analysis

The IBM SPSS version 25 (Armonk, New York, USA) was used for statistical analysis. Normal distribution of continuous data was assessed by visual inspection (histogram, qq-plot, boxplot) and by descriptive characteristics (mean, median, kurtosis, skewness). The χ^2^-test and Fisher’s exact test (if more than 25% of the cells had expected counts < 5) were used for categorical variables to test for significant differences between groups. A one-factorial analysis of variance (ANOVA) was performed for ordinally scaled variables. The *t*-test was used to compare continuous values between two groups (normal distributed variables, i.e., age). The Wilcoxon–Mann–Whitney U test was used for non-normally distributed parameters (preoperative SAA, SAA on day 10).

Two multivariable logistic regression models were applied to investigate the influence of enterotomy, resection and study group with respect to the probability to develop SSI within 10 days/30 days after surgery, respectively. Odds ratios (OR), along with their 95% confidence intervals (95% CI), were calculated whenever possible. *p*-values < 0.05 were considered significant. 

## 3. Results 

### 3.1. Study Population

Here, we report on 67 horses which fulfilled the inclusion criteria for this study, with 30 horses belonging to the SSG and 37 to the 5DG. A detailed overview of each horse’s gender, breed, age and kind of abdominal surgery is provided in Supplemental Table S1. There was no significant difference regarding the distribution of horse breeds between the study groups (*p* = 0.09, Fisher’s exact test). Six horses were stallions (all 5DG), 31 geldings (16, SSG; 15, 5DG) and 30 mares (14, SSG; 16, 5DG). There was no significant difference with respect to gender between the groups (*p* = 0.06, Fisher’s exact test).

### 3.2. Postoperative Events

A total of three horses (1 SSG; 2 5DG) underwent relaparotomy within 3–10 days after surgery, and one of them was euthanised (5DG) because a bladder carcinoma was suspected. A further five horses were euthanised (2 SSG; 3 5DG) within 3–10 days after the surgical intervention. None of the horses enrolled in this study were euthanised because of an adverse event possibly associated with the antibiotic regimen, especially SSI, colitis or haemolytic anaemia. Three horses were discharged before the tenth day after surgery (1 SSG; 2 5DG). Information about six horses (5 SSG; 1 5DG) was not achieved by follow-up telephone calls. 

### 3.3. Adverse Events Following Colic Surgery Which Were Probably Associated with the PAP Regimen

As a result, 8 of 30 (26.6%) horses belonging to the SSG and 7 of 37 (18.9%) equine patients of the 5DG showed an adverse event following colic surgery. Since the limit was set at 15%, the difference of 7.7% indicates non-inferiority of the single-shot antibiotic regimen (study power: 60%). A comprehensive overview of all SSI cases together with adverse events possibly associated with the PAP regimen is provided in Table 1.

Altogether, 4 of 30 horses belonging to the SSG (13%) and 1 of 37 horses belonging to the 5DG (3%) developed a SSI within 10 days after surgery (Table 2), a difference that lacked significance (*p* = 0.2, Fisher’s exact test).

Considering the onset of SSI within 30 days after surgery, one horse (SSG) that was hospitalised for more than 10 days developed a purulent infection on day 12. Another horse (SSG) was presented to the clinic because of SSI on day 15 following surgery. Two further horses (1 SSG; 1 5DG), for which microbiological culture results were unfortunately not available, developed SSI within 30 days after surgery (Table 2). 

Again, the recorded cases of SSI which occurred within 30 days after surgery lacked statistical significance between the SSG and the 5DG (*p* = 0.07, Fisher’s exact test). Incisional hernia was reported for one case (SSG). However, the specific day of the incident was not available.

Results recorded for horses with and without enterotomy and/or resection did not differ significantly considering the development of SSI during the first 10 days after surgery (multivariable logistic regression, global *p* = 0.1). However, analysing SSI that occurred within 30 days after surgery (data obtained by follow-up calls), horses that required enterotomy almost significantly more often developed SSI than horses without enterotomy (*p* = 0.051, OR = 15.4, 95% CI 0.99–240, multivariable regression, global *p* = 0.03). Furthermore, there was no significant difference in the development of both SSI within 10 (*p* = 0.503, chi squared test) and 30 days (*p* = 0.583, chi squared test) between clean and clean-contaminated procedures (Table 2).

Data available on horses belonging to the SSG and the 5DG included age (13.5 ± 7.6 years versus 13.6 ± 7.7 years; *p* = 0.5, *t*-test), duration of general anaesthesia (2.9 ± 2.3 h versus 2.9 ± 2.3 h, *p* = 0.6, Wilcoxon–Mann–Whitney U test) and hospitalisation time (12.7 ± 4.5 days versus 12.6 ± 4.5 days, *p* = 0.6, *t*-test), and lacked significant differences. Details for each horse are presented in Supplemental Table S1.

The probable cause of the abdominal disorders associated with colic was most often localised in the large intestine (*n* = 37), followed by dysfunction of the small intestine (*n* = 21). Both intestine parts seemed to be affected in eight horses, and a further horse suffered from a diaphragmatic hernia (all details are provided in Supplemental Table S1). The study groups investigated here (SSG versus 5DG) lacked significant differences regarding the location (large intestine/small intestine/both) of the intestinal disorder (*p* = 0.8, Fisher’s exact test). Furthermore, there was no significant difference between the groups regarding classification, according to Cruse and Foord [3], of the surgery (clean: 12 SSG, 9 5DG; clean-contaminated: 18 SSG, 28 5DG; *p* = 0.19). 

Enterotomy was performed in 39 horses (17 SSG; 22 5DG; *p* = 0.8, chi squared test) and intestinal resection in 12 horses (1, SSG; 11, 5DG; *p* = 0.005, OR 12.269, chi squared test). Thus, there was no difference regarding the necessity for enterotomy between the groups, but horses assigned to the 5DG needed a resection significantly more often.

Besides SSI, adverse events which are commonly associated with the administration of antibiotics in horses were recorded. Five horses developed postoperative colitis, four of them were assigned to the 5DG and one to the SSG (*p* = 0.2, Fisher’s exact test, OR 3.52, 95% CI 0.37–33.26). Penicillin, gentamicin and non-steroidal anti-inflammatory drugs (NSAID) were discontinued, and the horses received fluids, probiotics and metronidazole. *Clostridium perfringens* was detected using microbiological cultures in two faecal samples obtained from these horses (Table 1).

In addition, clinical signs of haemolytic anaemia have been perceived in three further cases (all 5DG; *p* = 0.1). An abrupt cessation of penicillin and gentamicin administration was followed by a restored physiological haematocrit in all cases. None of these patients required blood transfusion. For postoperative adverse events, the classification (clean versus clean-contaminated) lacked statistical significance (colitis: *p* = 0.142, chi squared test, haemolytic anaemia: *p* = 0.317, chi squared test). 

### 3.4. Bacteria Associated with SSI

Bacteria isolated from samples of SSI (Table 1) included common enteric bacteria such as *Enterococcus faecium*, *Escherichia coli*, *Enterobacter cloacae*, *Klebsiella pneumoniae* and *Enterbacter aerogens*. Most of these bacteria exhibited antimicrobial resistance towards more than four different antibiotic classes, justifying their classification as MDR. In addition, three wound swabs were also positive for MRSA.

### 3.5. Laboratory Tests

We monitored the white blood-cell count, SAA and fibrinogen of horses belonging to both study groups. Mean values, standard deviation/median and the range of non-normally distributed values are displayed in Table 3. Some samples were not available due to euthanasia, early hospital discharge and/or management errors during emergency shifts. The study groups lacked significant differences regarding white blood-cell count, SAA and fibrinogen.

## 4. Discussion

This study was designed as a randomised pilot study aimed to test for non-inferiority of the “single-shot” PAP to the conventional “5-day lasting” antibiotic regimen considering SSI and other adverse effects (i.e., colitis and haemolytic anaemia). Since the 15% non-inferiority margin was not exceeded, the primary aim of the study, demonstrating the non-inferiority potential of the single-shot regimen, was achieved. However, caution is needed with respect to the interpretation of the results presented here, since a rather limited study power (60%) prohibits general conclusions. While further research on the subject in terms of clinical trials is clearly needed, the overall sample sizes available are always limited by the equine patients actually needing colic surgery. A study power of 80%, for instance, would require 50 horses per group (total: 100), a number which likely requires a study period of three years or even more considering our inclusion criteria.

Since most colic surgeries are classified as clean-contaminated [17], horses subjected to colic surgery should receive PAP. At present, studies reporting on peri- and post-operative use of antibiotics in equine laparotomy are scarce. A study by Durward-Akhurst et al. did not find a significant difference in incisional complications comparing a 72 to a 120 h-lasting antibiotic regimen [17]. Similarly, we could not identify any beneficial effects of a prolonged administration of antibiotics after colic surgery.

### 4.1. Surgical Site Infections Following Surgery 

The onset of SSI after laparotomy commonly occurs between the 4th and 14th day after surgery [30,31,32,33], which is mostly within the typical 10-day hospitalisation period. Additionally, a follow-up call, which was admittedly late in many patients, was used to identify patients that developed SSI within 30 days after colic surgery. Surgical procedures, general anaesthesia, PAP application and monitoring of the patients following surgery had been well-standardised due to the study design.

The total SSI rate in the 5DG was 5.4%, and 23.3% in the “single-shot” group (SSG). Previous research reported SSI rates between 15% and 50% after colic surgery [6,17,20,21,22,23]. The PAP regimens used in these studies differed, but most authors reported administration of a combination of penicillin and gentamicin beyond the immediate 24 h. Even though SSI occurred more often in horses belonging to the SSG, the results lacked significant differences. However, the SSI rate of the SSG did not exceed the SSI rates reported for colic surgery in the literature [6,20,21,22,23,24,34].

### 4.2. Surgery-Associated Factors

The origin of the intestinal disorder, causing colic, was hypothesised to influence incisional wound healing. A previous study identified large intestinal obstruction as a risk factor and suspected exteriorisation of the large intestine to cause trauma to the incision [35]. However, other authors did not support this hypothesis [36]. Furthermore, small intestinal lesions and the necessity for anastomosis were both suspected to be risk factors for SSI [37]. Similar to Mair et al. [5], who could not confirm the hypothesis that large intestinal lesions predispose to incisional complications, an association between the (most probable) origin of the gastrointestinal pain and the development of SSI after colic surgery was not noticeable. 

According to Cruse and Foord [2], who classified surgical wounds in humans, to open a hollow organ such as the intestine is always classified as a clean-contaminated procedure and, therefore, associated with an increased SSI risk. Accordingly, laparotomy without enterotomy and/or resection was classified as “clean surgery” and laparotomy including enterotomy and/or resection was considered as “clean-contaminated surgery”. It seems likely that clean-contaminated operative interventions are associated with an increased risk of SSI in horses [38,39] but the studies available did not support this view [35,36,37,40,41,42]. Enterotomy and/or resection was not associated with increased SSI rates in this study, irrespectively of the antibiotic regimen the horse was assigned to.

### 4.3. Bacteria Associated with SSI in Horses Receiving Colic Surgery

Gram-positive and -negative bacteria residing on the horse’s skin and mucosal surfaces are among the common causes of SSI [13]. Previous research on MDR-colonised horses showed that 10.7% of the horses entering a clinical setting carried ESBL-producing *E. coli* [29] and 3.5% MRSA [28]. Consequently, shielding the wound from further damage, especially an infection caused by MDR bacteria residing on the patient and/or the clinical environment, is crucial. Incoming colonised horses may spread the MDR bacteria, and other animals or even humans can become colonised [43]. Once these bacteria cause an SSI, it is often difficult to treat, since the remaining therapy options are limited [43,44,45,46,47]. In light of the MDR bacteria associated with SSI identified in this study (Table 1), enhanced protection of the incisional wound seems beneficial: Since a previous study reported a reduction of 45% considering incisional complications by use of abdominal bandages [33], it seems likely that prolonged application supports wound preservation. However, more research on this subject is needed.

A significant reduction of SSI was achieved by placing a stent bandage (a sterile cover sewn over the incision), which was replaced by an abdominal bandage when the horse has recovered from anaesthesia [23].

To summarise, our results once more underline the importance of adequate hygiene management in equine clinics, including personal and hand hygiene, patient hygiene and implementation of proper cleaning/disinfection protocols, as described by several publications [9,48,49].

### 4.4. Postoperative Colitis

Five horses suffered from postoperative colitis, and four of them belonged to the 5DG. Postoperative colitis is a common complication after colic surgery and may be influenced by the nature of the intestinal lesion, alterations in gut motility, surgical manipulation of the intestines and antibiotics disrupting the physiological gastrointestinal bacterial ecosystem [50,51,52]. The reasons for colitis following administration of antibiotics are multifactorial, but an important factor is the unavoidable change of the structure and composition of the gastrointestinal microbiota, which is particularly associated with the administration of antibiotics [53,54].

In particular, NSAIDs are also known to be the cause of (postoperative) colitis. These drugs are not only associated with gastric ulceration but also with right dorsal colitis and a delayed mucosal healing [55,56,57,58,59]. The five horses suffering from postoperative colitis received a standard course of NSAIDs (5 days, 1.1 mg/kg flunixin-meglumine BID) after surgery. Previous studies identified horses suffering from large intestinal lesions being associated with an increased risk of postoperative colitis, while detection of *Clostridia* spp. and *Salmonella* seemed of lesser importance [60,61]. Only 2/5 faecal samples from horses with colitis were culture-positive for *Clostridium perfringens*. The causes of postoperative colitis in the remaining patients were possibly associated with a combination of the factors mentioned above, since most of the horses had primary large intestinal lesions (Table 2) and received antibiotics for 5 days after surgery and NSAID. Since four out of the five patients that developed postoperative colitis received the prolonged PAP regimen, further research on the particular effects of PAP on the equine intestinal microbiome is warranted.

### 4.5. Haemolytic Anaemia

Three horses developed haemolytic anaemia. Penicillin-induced immune-mediated haemolytic anaemia is a well-described phenomenon in equine literature and is considered as a severe clinical complication associated with prolonged administration of the antibiotic. The proposed pathomechanism causing haemolysis is associated with the interaction of anti-penicillin IgG antibodies with penicillin-coated equine erythrocytes [62,63,64,65]. Of note, only horses belonging to the 5DG have developed haemolytic anaemia, although the difference is lacking significance. Since all horses have recovered quickly after suspension of further antibiotic courses, administration of penicillin seems to be the most likely cause for the anaemic condition of these patients.

Heparin is commonly used to treat haemostatic abnormalities in horses suffering from severe gastrointestinal diseases, septicaemia and endotoxemia [66]. The horses in the present study received either un-fractioned or low-molecular heparin. Heparin may cause a regenerative, macrocytic anaemia and extravascular haemolysis by opsonisation of erythrocytes, which induces their early phagocytosis [67,68]. Other theories suspected that heparin may act as a hapten for antibody-mediated erythrocyte agglutination [69], similar to the pathomechanisms discussed for human immune-mediated thrombocytopenia [70]. A further hypothesis is that a decreased plasma proteolytic activity allows for protein adherence to red blood cells, followed by their agglutination [71]. 

## 5. Conclusions

Perioperative prophylaxis in terms of a single-shot antibiotic regimen has the potential to be non-inferior to the commonly used antibiotic regimen lasting five days following colic surgery. Further clinical trials on the subject are needed.

## Figures and Tables

**Table 1:** Adverse events following colic surgery possibly associated with the antibiotic regimen (p.o. = postoperatively).

				Surgical Site Infections (SSI)		
Group	ID	Diagnosis during Surgery	Adverse Event after Surgery *	10 Days	30 Days	Results of Microbiological Cultures from SSI
SSG	10	Torsio coli	SSI	−	+	*E. faecium*
	13	Ileal obstipation	SSI	+	+	*E. cloacae* (ESBL)
	19	Ileal obstipation	SSI	+	+	MRSA, *E. coli*, *Fusobacterium* spp., *B. fragiles*
	23^a^	Caecal obstipation	Colitis	−	−	-
	28	Nephrosplenic entrapment	SSI	−	+	*E. coli, K. pneumoniae,* MRSA, *E. faecium*
	29	Lipoma pendulans	SSI	+	+	*E. coli, E. cloacae, E. faecium*
	46	Obstipation of the ascending colon	SSI	−	+	n.a.
	66	Lipoma pendulans	SSI	+	+	*E. cloacae*
5DG	12	Obstipation of the ascending colon	Colitis	−	−	-
	18	Incarceration of the ascending colon	Haemolytic anaemia	−	−	-
	33	Torsio caeci with incarcerated jejunum	Colitis	−	−	-
	41	Lipoma pendulans	SSI	+	+	*E. aerogenes* (ESBL), MRSA
	56 ^a^	Meteorism of the colon	Colitis	−	−	-
	60	Diaphragmatic hernia	Haemolytic anaemia	−	−	-
	62	Lipoma pendulans	Colitis, Haemolytic anaemia	−	+	n.a.

The table shows adverse events noted for horses belonging to the SSG and those recorded for horses which received antibiotics at 5 days (5DG) after surgery. Abbreviations: ID, individual horse number (also see Supplemental Table S1), 10 days, onset of SSI within 10 days following surgery; 30 days, SSI developed within 30 days after surgery; *, ≥3 days after surgery; +, horse developed SSI; −, horse did not develop SSI. ^a^: horse with colitis and *Clostridium perfringens*-positive faecal sample; *E. faecium*, *Enterococcus faecium*; *E. cloacae*, *Enterobacter cloacae*; MRSA, methicillin resistant *Staphylococcus aureus*; *E. coli*, *Escherichia coli*; *B. fragiles*, *Bacteriodes fragiles*; *E. aerogenes*, *Enterobacter aerogenes*; *K. pneumoniae*, *Klebsiella pneumoniae*; ESBL, extended-spectrum beta-lactamase-producing.

**Table 2:** Comprehensive overview of study groups and study outcomes.

	Single-Shot Group		5-Day Group		
	*n* = 30		*n* = 37		*p*
	*n*	%	*n*	%	
**Surgical site infections (SSI)**					
SI within 10 days after surgery	4	13	1	3	0.17
**total SSI**	7	23	2	5	0.07
**Classification of surgery**					
clean	12	40	9	24	0.13
clean-contaminated	18	60	28	76	0.13
contaminated	0	0	0	0	
**Colitis**	1	3	4	11	0.37
**Haemolytic anaemia**	0	0	3	8	0.25

**Table 3:** Mean and standard deviation/median and of the laboratory parameters examined.

			SSG		5DG		
Parameter	Day	Time	*n*	Mean ± Standard Deviation or Median (Min–Max)	*n*	Mean ± Standard Deviation or Median (Min–Max)	*p*
**WBC (G/L)**	0	variable	30	8.57 ± 2.36	37	9.22 ± 3.68	0.399
	1	morning	30	6.81 ± 4.21	37	5.77 ± 2.23	0.202
		evening	30	6.36 ± 3.94	37	6.34 ± 2.61	0.957
	2	morning	30	5.36 ± 3.45	37	5.47 ± 2.64	0.886
		evening	29	4.96 ± 3.24	33	5.24 ± 2.4	0.692
	3	morning	29	5 ± 3.06	37	5.17 ± 2.47	0.796
		evening	26	5.15 ± 2.9	34	5.48 ± 2.51	0.637
	4	morning	27	5.45 ± 2.72	36	5.36 ± 2.05	0.873
		evening	25	6.19 ± 2.79	32	5.69 ±1.68	0.407
	5	morning	27	6.91 ± 2.36	35	6.94 ± 2.14	0.953
		evening	25	7.71 ± 2.17	29	8.01 ± 20.8	0.603
	10	morning	25	10.59 ± 3.78	29	10.3 ± 3.74	0.774
**SAA (µg/mL)**	0	variable	22	13.3 (3.5–748.68)	30	18.4 (3.5–649.36)	0.541
	1	morning	28	598.81 ± 163.11	37	602.73 ± 204.1	0.934
	2	morning	28	743.71 ± 121.51	33	750.1 ± 196.57	0.882
	3	morning	28	781.98 ± 137.87	35	759.57 ± 136.4	0.521
	4	morning	26	705.72 ± 167.22	32	732.48 ± 196.82	0.557
	5	morning	26	632.55 ± 197.15	29	629.34 ± 226.04	0.956
	10	morning	25	110.82 (5.43–783.05)	26	64.93 (3.5–707.62)	0.356
**Fibrinogen (mg/dL)**	0	variable	27	188.63 ± 70.52	31	199.26 ± 51.34	0.51
	1	morning	30	225.48 ± 72.112	35	231.1 ± 60.35	0.733
	2	morning	30	564.86 ± 59.45	34	274.35 ± 63.43	0.54
	3	morning	29	270.51 ± 81.81	34	319.19 ± 167.87	0.16
	4	morning	27	288.43 ± 72.37	33	306.48 ± 65.79	0.316
	5	morning	26	288.29 ± 69.15	32	309.64 ± 76.1	0.273
	10	morning	25	285.55 ± 79.77	25	265.36 ± 89.87	0.405

## Data Availability

The data presented in this study are available in the article and Supplemental Table S1.

## Supplementary Materials

The following are available online at https://www.mdpi.com/article/10.3390/antibiotics10050587/s1, Table S1: Details of horses participating in the study.
