# Supplementary material for: A Pilot Randomised Clinical Trial Comparing a Short-Term Perioperative Prophylaxis Regimen to a Long-Term Standard Protocol in Equine Colic Surgery
Source: Antibiotics (Basel). 2021 May 16;10(5):587. doi: 10.3390/antibiotics10050587 (PMC8156649; doi:10.3390/antibiotics10050587)
Supplement: Supplementary file 1 [file antibiotics-10-00587-s001.zip › antibiotics-1148483-supplementary.pdf]

Number	Antibiotics	Date of surgery	Relaparotomy	Gender	Age (years)
1	5 days	2018/1/10	no	Mare	11
2	single shot	2018/1/29	no	Mare	4
3	single shot	2018/2/3	no	Gelding	7
4	single shot	2018/2/3	no	Mare	16
5	5 days	2018/2/16	no	Mare	19
6	5 days	2018/4/2	no	Stallion	5
7	single shot	2018/4/20	no	Gelding	8
8	single shot	2018/4/26	no	Mare	7
9	5 days	2018/5/27	no	Mare	11
10	single shot	2018/7/5	no	Mare	5
11	5 days	2018/7/26	yes	Gelding	8
12	5 days	2018/8/5	no	Stallion	7
13	single shot	2018/8/15	no	Gelding	13
14	5 days	2018/9/10	no	Stallion	24

15	5 days	2018/9/13	no	Gelding	15
16	5 days	2018/9/27	no	Gelding	24
17	single shot	2018/10/1	no	Gelding	24
18	5 days	2018/10/9	no	Mare	15
19	single shot	2018/10/13	no	Mare	20
20	single shot	2018/11/1	no	Gelding	9
21	single shot	2018/11/4	no	Gelding	4
22	single shot	2018/11/17	yes	Gelding	9
23	single shot	2018/11/26	no	Gelding	25
24	single shot	2019/1/3	no	Gelding	9
25	5 days	2019/1/6	no	Gelding	4
26	single shot	2019/1/27	no	Mare	15
27	single shot	2019/2/4	no	Mare	6
28	single shot	2019/2/10	no	Gelding	11
29	single shot	2019/2/11	no	Gelding	20
30	5 days	2019/2/14	yes	Gelding	13
31	single shot	2019/2/16	no	Gelding	22
32	5 days	2019/3/4	no	Mare	22
33	5 days	2019/3/19	no	Mare	23
34	5 days	2019/3/31	no	Gelding	23
35	5 days	2019/4/17	yes	Gelding	27
36	5 days	2019/4/20	no	Mare	17
37	5 days	2019/5/2	no	Mare	16
38	5 days	2019/5/6	no	Mare	26

39	5 days	2019/5/23	no	Mare	9
40	single shot	2019/5/27	no	Gelding	13
41	5 days	2019/7/4	no	Gelding	19
42	5 days	2019/7/6	no	Mare	14
43	5 days	2019/7/11	no	Mare	4
44	5 days	2019/7/21	no	Stallion	0.42
45	5 days	2019/7/23	no	Gelding	29
46	single shot	2019/7/29	no	Mare	10
47	5 days	2019/8/3	no	Stallion	12
48	5 days	2019/8/7	no	Gelding	20
49	5 days	2019/8/11	no	Mare	12
50	single shot	2019/8/15	no	Mare	13
51	single shot	2019/9/5	no	Mare	6
52	5 days	2019/9/11	no	Gelding	16
53	single shot	2019/9/26	no	Mare	21
54	single shot	2019/10/22	no	Gelding	15
55	single shot	2019/11/4	no	Gelding	9
56	5 days	2019/11/9	no	Mare	1
57	single shot	2019/11/9	no	Mare	14
58	5 days	2019/12/14	no	Mare	0.58
59	5 days	2020/1/5	no	Gelding	20
60	5 days	2020/1/7	no	Stallion	2
61	5 days	2020/1/19	no	Gelding	8
62	5 days	2020/1/21	no	Mare	unknown
63	single shot	2020/1/31	no	Gelding	7
64	5 days	2020/2/11	no	Gelding	6
65	single shot	2020/2/14	no	Mare	17
66	single shot	2020/2/19	no	Mare	27

67	5 days	2020/2/24	no	Gelding	30

Breed	Classification of surgery	Localisation of the surgical lesion	SSI during the first 10 days post operatively	SSI total
Quarab	clean	Large intestinal	no	no
Mecklenburge r	clean-contaminated	Small intestinal	no	no
Hanoverian	clean	Small intestinal	no	no
Trakehner	clean-contaminated	Large intestinal	no	no
Brandenburge r	clean-contaminated	Large intestinal	no	no
German Sports Horse	clean	Small intestinal	no	no
German Sports Horse	clean	Small intestinal	no	no
Polish Warmblood	clean-contaminated	Large intestinal	no	lost to follow up
German Sports Horse	clean-contaminated	Large intestinal	no	no
Thoroughbred	clean-contaminated	Large intestinal	no	yes
American Quarter Horse	clean-contaminated	Small and large intestinal	No	yes after relaparot omy
Warmblood	clean-contaminated	Large intestinal	no	no
Hanoverian	clean-contaminated	Small intestinal	yes	yes
Shettland Pony	clean-contaminated	Large intestinal	no	no

Warmblood	clean-contaminated	Large intestinal	no	no
Konik	clean-contaminated	Large intestinal	no	no
Shettland Pony	clean-contaminated	Large intestinal	no	no
German Riding Pony	clean-contaminated	Large intestinal	no	no
Hanoverian	clean-contaminated	Small intestinal	yes	yes
Thoroughbred	clean-contaminated	Large intestinal	no	no
American Miniature Horse	clean-contaminated	Large intestinal	no	no
Oldenburger	clean-contaminated	Small intestinal	no	no
Haflinger - Cross	clean-contaminated	Large intestinal	no	no
Holsteiner	clean-contaminated	Large intestinal	no	lost to follow up
German Sports Horse	clean-contaminated	Large intestinal	no	no
Pony - Cross	clean	Large intestinal	no	no
German Sports Horse	clean	Small intestinal	no	lost to follow up
German Sports Horse	clean	Large intestinal	no	yes
Trakehner	clean	Small and large intestinal	yes	yes
Mecklenburge r	clean-contaminated	Small intestinal	no	no
Sachsen - Anhaltiner	clean	Small intestinal	no	no
Bavarian Warmblood	clean-contaminated	Large intestinal	no	no
Brandenburge r	clean-contaminated	Small and large intestinal	no	no
Brandenburge r	clean-contaminated	Small and large intestinal	no	no
Icelandic horse	clean	Small intestinal	no	no
American Quarter Horse	clean-contaminated	Large intestinal	no	no
Haflinger	clean-contaminated	Small intestinal	no	no
Shettland Pony	clean-contaminated	Large intestinal	no	no

Shettland Pony	clean-contaminated	Large intestinal	no	no
German Sports Horse	clean	Large intestinal	no	no
Paint Horse	clean-contaminated	Small intestinal	yes	yes
Warmblood	clean-contaminated	Large intestinal	no	no
Norwegian Fjoord Horse Cross	clean	Small intestinal	no	no
Warmblood	clean-contaminated	Small intestinal	no	no
Arabian Horse	clean	Large intestinal	no	no
Lewitzer	clean-contaminated	Large intestinal	no	yes
German Riding Pony	clean-contaminated	Large intestinal	no	no
German Riding Horse	clean	Small intestinal	no	no
Standardbred-Cross	clean	Small intestinal	no	lost to follow up
Pony	clean-contaminated	Small intestinal	no	no
Hanoverian	clean-contaminated	Large intestinal	no	lost to follow up
Mecklenburger	clean-contaminated	Large intestinal	no	no
Shettland Pony	clean-contaminated	Small intestinal	no	no
Friesian	clean-contaminated	Large intestinal	no	lost to follow up
American Quarter Horse	clean	Small and large intestinal	no	no
Noriker	clean-contaminated	Large intestinal	no	no
Haflinger	clean-contaminated	Large intestinal	no	lost to follow up
Haflinger	clean	Small intestinal	no	no
Brandenburger	clean	Small intestinal	no	no
Traber	clean-contaminated	diaphargmatic hernia	no	no
German Sports Horse	clean-contaminated	Large intestinal	no	no
Draft Horse	clean-contaminated	Small intestinal	no	yes
Thoroughbred	clean	Large intestinal	no	no
Oldenburger	clean-contaminated	Large intestinal	no	lost to follow up
Warmblood	clean	Large intestinal	no	no
Thoroughbred	clean	Small intestinal	yes	yes

Norwegian Fjord Horse	clean-contaminated	Large intestinal	no	no

Results of the microbiological examination of the swab	Colitis	Hemolysis	WBC pior to sutgery (x10^3/µl)	SAA prior to surgery (µg/ml)	Fibrinogen prior to surgery (mg/dl)
	no	no	6.92	127.91	168
	no	no	10.48	748.68	184
	no	no	8.21		445
	no	no	8.88		218
	no	no	5.93	3.5	139
	no	no	9.62	36.24	217
	no	no	5.68	3.5	162
	no	no	12.01	3.5	172
	no	no	10.75	7.13	189
Enterococcus faecium, Staphylococcus epidemicus (methicillin resistant)	no	no	10.74	4.7	96.54
SBL-producing Enterobacter cloaceae, Enterococcus faecalis, Klebsiella pneumoniae, E. coli, Pseudomonas aeruginosa	no	no	7.48	37.87	167
	yes	no	5.87	7.28	355
ESBL-producing Enterobacter cloacae	no	no	10.87	6.39	217
	no	no	16.74	136.57	235

	no	no	2.92	69.27	189
	no	no	9.21	7.97	235
	no	no	7.83		128
	no	yes	12.82	3.5	226
MRSA, Edvherichia coli, Fusobacterium spp., Bacteroides fragiles	no	no	6.93	317.33	167.42
	no	no	7.02	4.19	211
	no	no	9.34	146.98	171
	no	no	10.27		116
	yes	no	7.84		261
	no	no	8.44	35.65	128
	no	no	5.21	470.45	275
	no	no	5.81	14.34	135
	no	no	11.47	11.39	135
	no	no	6.7	12.3	188
Escherichia coli, Enterobacter cloacae, Enterococcus faecium	no	no	13.66	9.66	203
	no	no	9.25	11.94	93.33
	no	no	9.8		131
	no	no	9.05	7.4	171
	yes	no	13.21	19.01	183
	no	no	7.3		
	no	no	14.58	30.47	183
	no	no	8.43	18.5	145.81
	no	no	10.04	24.54	177
	no	no	2.12	514.65	202

	no	no	6.33	3.5	226
	no	no	9.67	4.37	195
	no	no	18.97	9.24	308
	no	no	6.55	96.52	226
	no	no	3.07	3.5	182
	no	no	12.75	67.34	120
	no	no	8.26	4.28	209
	no	no	11.76	3.56	134
	no	no	10.88	5.01	188
	no	no	10.79	649.36	149.07
	no	no	3.8		
	no	no	8.76	4.18	195
	no	no	8.16		
	no	no	10.43	15.3	218
	no	no	5.28	126.15	236
	no	no	6.98	14.46	300
	no	no	8.5	112.65	246
	yes	no	8.75		
	no	no	2.6		
	no	no	9.36		
	no	no	11.64		
	no	yes	13.46		218
	no	no	11.75		209
	yes	yes	9.88	12.16	
	no	no	7.43	35.56	
	no	no	8.64	18.29	182
	no	no	6	20.57	184
	no	no	9.91	14.73	134

	no	no	8.62	24.49	192

WBC day 1 morning (x10^3/µl)	SAA day 1 (µg/ml)	Fibrinogen day 1 (mg/dl)	WBC day 1 evening (x10^3/µl)	WBC day 2 morning (x10^3/µl)	SAA day 2 (µg/ml)	F1 fibrinogen day 2 (mg/dl)
4	647.4	220	3.53	2.63	800.33	251
10.64	964.42	240	9.86	9.42	987.13	299
4.16	788.61	212	3.88	3.4	831	212
8.99	735.76	240	7.5	5.34	875.01	278
6.13	837.41	235	6.05	5.09	896.5	256
1.43	727.6	324	2.01	2.03	1011.8	324
6.66	259.83	143	1.59	2	651.54	209
6.53	724.77	245	6.44	6.57	809.07	269
7.28	931.21	226	10.18	7.73	983.82	297
2.63	584.71	79.22	3.46	5.29	807.61	160.39
6.89	637.6	245	6.47	2.56	717.05	314
3.14	374.19	189	4.07	5.05	822.07	290
10.58	555.73	226	9.29	6.52	732.98	282
2.93	542.11	245	8.03	7.36	740.05	314

4.88	657.26	269	3.41	3.17	648.23	249
6.06	701.98	163.5	7.25	8.5		196
5.44	446.29	256	4.92	4.52	649.81	194.5
8.23	708.45	138.5	5.74	5.68	764.42	163.8
2.8	714.96	220.07	4.49	3.7	724.11	367
4.3	437.81	211	3.81	3.91	1043	228
4.63	752.1	290	5.44	4.77	772.36	270
12.25		306	9.68	5.19		249
23.59	502.79	249	23.8	20.49	615.21	325
6.43	651.62	238	5.57	4.83	666.22	282
5.41	563.71	325	4.83	4.26	952.98	308
3.5	343.31	203	5.5	4.99	735.23	261
7.25	661.07	189	7.04	3.14		246
3.47	588.91	196	3.45	2.45	652.26	238
12.79	671.05	290	7.76	4.92	721.25	153.82
3.91	428.23	96.56	6.4	4.6	658.04	104.79
3.16	790.89	128.97	3.71	4.42	842.11	249
4.02	785.12	249	3.89	4.33	652.18	275
6.23	735.14	317	10.62	10.76	757.59	238
3.48	787.78	178.75	4.91	4.6	1108.4	206.19
8.58	710.46	211	9.52	7.13	835.12	236
7.29	721.68	370	8.64	4.81	743.79	317
6.17	392.38	218	7.15	4.26	645.88	236
2.25	724.97	172.29	3.41	3.69	860.2	317

7.35	269.13	284	10.58	6.92	721.05	300
6.07	565.66	236	3.59	2.61	618.44	258
10.16	208.31	258	12	11	591.41	317
5.22	547.02	326	1.97	1.38		226
7.34	637.25	270	7.3	7.52	686	308
6.22	812.34	258	3.34	3.2	884.56	411
12.16	228.18	218	6.91	4.54	672.38	281
3.82	301.58	177	4.05	7.87	569.92	284
6.91	727.04	127.95	10.71	12.26	600.08	300
6.05	518.53	236	7	4.69	874.73	317
5.38	641.79	226	4.96	4.37	469.34	270
7.15	630.22	246	5.16	4.54	644.1	258
6.16	654.69	484	4.84	5.88	911.97	358
7.76	691.37	202	9.88	8.85	11.98	370
6.3	497.12	182	5.1	1.48	628.36	236
9.06	676.32	300	8.45	7.02	777.61	445
6.64	477.1	258	9.17	5.97	633.7	317
4.89	563.69	218	6.25	9.5	822.75	308
2.03	800.13	246	4.08	2.06	831.56	300
7.95	878.16		6.91	6.71	572.27	147.28
3.13	540.62	209	5.63	3.95		
7.47	866.32		7.96	5.61	935.33	
4.8	19.1	182	5.75	4.14	904.38	317
3.33	635.08	300	2.78	2.15	844.58	347
5.97		149	7.91	7.98	843.3	258
4.78	523.21	193	4.73	4.22		
6.45	511.42	176	3.95	2.65	630.86	214
4.76	477.74	148	7.19	6.85	618.2	245

4.44	379.2	188	6	3.02	563.96	216

WBC day 2 evening (x10^3/µl)	WBC day3 morning (x10^3/µl)	SAA day 3 (µg/ml)	Fibrinogen day 3 (mg/dl)	WBC day 3 evening (x10^3/µl)	WBC day 4 morning (x10^3/µl)	SAA day 4 (µg/ml)
2	3.64	822.64	283	3.43	3.83	787.48
10.93	10.44	954.33	326	7.92	8.07	890.35
2.02	2.68	831.04	212	2.8	4.21	741.46
4.75	3.49	872.45	240	1.17	2.89	807.51
4.36	5.15	983.66	282	5.22	4.74	964.86
3.04	4.17	1014.3	344	4.1	4.85	1026.1
1.14	1.94	743.35	269			
5.15	4.03	767.06	282	4.25	3.88	673.57
8.72	6.36	973.3	206.13	5.8	6.28	951.15
5.59	5.09	904.48	224.02	4.73	5.54	
2.3	1.78	748.77	282	4.9	6.01	668.75
4.77	4.76	834.34	324	4.49	5.15	
6.56	4.2	665.66	269	4.23	3.69	582.29
9.28	5.99	768.3	333	6.7	5.62	714.67

4.17	1.59	607.29	245	4.09	3.88	585.45
6.69	4.89	826.57	222.52	4.4	4.18	781.27
3.75	2.76	690.1	355	4.67	3.95	570.36
8.74	7.26			6.85	4.92	
3.8	3.36	700.84	394	7.28	7.44	768.57
4.06	4.66	810.45	275	4.6	4	1013.5
4.23	4.33	732.24	186.54	4.32	5.54	633.05
3.47						
18.54	17.65		371	16.67	17.07	203.09
3.9	3.94	658.8	249	3.42	3.54	624.58
4.3	4.12	584.7	336	4.58	5.15	379.87
5.11	4.83	1039.7	290	3.9	4.47	976.13
2.6	3.88	1121.2	298	3.78	5.99	794.88
3.02	3.77	645.32	151.41	2.88	3.6	738.46
5.08	5	1026.9	169.77	6.06	5.47	725.29
3.77	3.9	841.38	162.39	3.82	3.6	918.37
3.42	5.31	783.57	275	6.16	7.16	787.67
4.28	4.71	830.71	316	6.15	5.61	1095.3
8.01	5.67	799.4	290	5.47	2.4	787.21
5.17	5.79	811.98	218.2	6.27	5.99	1056.7
4.72	4.5	706.98	258	3	3.54	681.96
7.98	8.02	744.52	306	8.8	6.38	798.23
4.08	3.33	658.96	163.1	3.69	2.85	620.62
4.12	4.74	670.99	317	5	6.11	605.21

6.04	6.16	731.86	336	5.54	4.74	711.01
2.89	3.03	624.17	326	3.99	5.22	583.9
10.18	7.33	836.17	326	11.96	6.6	640.07
1.89	3.15	857.41	195			
7.6	7.81	667.84	336	6.69	6.77	756.6
2.87	4.26	682.21	427	5.01	4.85	871.06
4.19	4.34	499.8	300	4.82	5.27	582.61
7.56	8.69	639.01	317	8.11	7.39	640.32
9.65	11.03	656.38	383	5.7	9.32	605.73
4.08	2.25	891.81	308	2.5	2.52	874.49
4.36	4.34	354.57	236	5.83	6.75	136.01
4.43	4.06	612.88	270	3.41	3.3	568.63
6.73	6.3	855.4	336	6.81	6.38	766.33
8.16	9.45	725.22	336	6.79	6.25	889.67
2	1.86	640.73	218			
5.79	7.52	695.76	465	6.99	6	737.57
4.64	4.2	660.74	284		5.68	682.93
	12.41	908.4		15.48	11	832.25
	4.43	775.57	270	4.43	4.25	455.89
	9.02	791.94	270		12	521.11
3.37	3.35	641.04	223	3.96	4.16	620.94
	2.83	928.7	347	3.7	4.4	677.92
	3.42		358		4.46	
3.28	2.85	663.56	383	4.2	4.98	635.64
4.43	4.03	883.15	258	3.35	3.46	859.32
3.69	3.97	769.88		3.82	4.16	
3.05	3.51	873.28	24	3.27	4.05	885.97
5.11	5.9	687.13	240	4.59	4.97	637.16

3.15	3.06	749.37	>1200	3.48	3.48	725.17

Fibrinogen day 4 (mg/dl)	WBC day 4 evening (x10^3/µl)	WBC day 5 morning (x10^3/µl)	SAA day 5 (µg/ml)	Fibrinogen day 5 (mg/dl)	WBC day 5 evening (x10^3/µl)	WBC day 10(x10^3/µl)
263	2.94	5.7	699.47	283	5.91	7.61
347	7.27	3.45	728.68	347	7.41	14.8
230	5.33	6.33		217	7.19	19.11
220	3.03	7.54	713.86	230	6.27	
282	5.08	7.09	970.34	297	8.41	10.88
367						
314	5.7	9.55	516.6	314	7.47	11.05
207.29	6.53	9.24		218	12.46	10.8
246	5.71	7.9	863.87	251.56	9.32	10.27
314	8.7	9.52	661.03	314	10.08	
344	6.07	8.6	766.13	367	6.19	9.16
409	2.92	6	707.35	333	7.59	8.18
306	6.28	6.4	596.53	297	9.14	9.78

269	5.69	7.59		238	8.5	7.38
228.21	4.11	6.83	702.55	560	7.45	6.23
180.9	4.35	4.4	409.93	172.22	4.6	8.51
	6.93	4.73	665.4	219.14	3.4	11.71
394	11.2	10.43	746.65	394	12.11	21.26
282	5.77	4.08	699.55	306	4.89	7.61
189.28	7.28	5.64	456.51	249	10.64	10.35
347	16.12	13.42	640.93	316	11.79	16.57
275	4.4	5.34	982.66	282	5.88	9.17
316	6.12	9.95	180.74	316	11.26	7.4
275	4.3	4.55	892.52	275	4.31	6.79
316	6	7.22	733.05	336	8.3	10.69
158.01	4.2	4.72	464.49	173.63	6.1	6.08
187.29	6	7.28	734.06	182.5	8.63	9.94
325	2.11	5.96	833.77	347	7.84	11.66
306	8.38	11.28	719.82	371	9.73	10.95
306	7.14	9.1	1016.5	325	11.6	
270	4.27	7.68	1081.9	258	9.4	8.6
217.38	5.96	5.65		217	7.19	
226	4.62	9.76	671.79	246	6.07	
325	6.14	6.6	527.73	317	7.25	11.27
385	2.96	2.69			5.92	8.81
326	7.57	8.55	625.01	187.39	9.37	11.87

336	7	9.1	547.73	308	7.75	9.53
336	8.34	7.4	704.54	358	7.07	6.93
358	6	5.48	797.27	370	10.67	14.04
326	7.12	8.62	506.01	308	9.87	9.71
484	3.99	5.06	630.31	463	6.12	16.63
326	6.58	5.38	335.77	308	6.7	9.4
336	7.19	6.58	602.22	147.73	7.83	13.15
173.81	8.65	8.29		284	9.73	10.85
300	2.63	2.54	799.17	336	6.84	5.91
236	7.29	8.97	15.17	258		
300	3.2	6.88	488.34	317	9.46	9.49
326	6.76	8.82	636.68	326	9.48	10.91
308	6.47	3.79	830.49	308	6.71	9.66
463	6.43	7.02	692.54	411	7.83	8.5
326		9.17	25.79			
284		10.38	588.43	308		25.6
270	5.42	6.26	296.72	300		8.42
284		9	466.07			
	5.88	6.31	496.98	252	6.8	9.79
433	5.65	4.8	592.53	418	8.08	11.08
370	5.03	3.96	633.05	358		7.58
383	6.93	6.66	583.74	383		7.1
238		5.44	790.64	308	5.54	6.97
	3.59	4.69			5.61	7.7
270	4.62	4.99	592.21	279	8.81	9.4
246	4.75	4.75	606.18	299	4.46	9.72

235		8.19	429.37	240		10.87

SAA day 10 (µg/ml)	Fibrinogen day 10 (mg/dl)
89.12	240
453.46	326
662.44	283
339.5	282
5.7	282
	160.31
780.14	245
139.38	344
731.88	409
104.31	297

3.5	235
118.52	203.21
5.43	166
130.14	359
783.05	400
110.82	211
456.51	211
504.56	347
230.23	261
40.73	306
17.6	261
32.93	152.71
12.63	249
121.69	347
707.62	347
243.76	400
22.38	174.78
24.75	152.81
8.23	184.76
15.69	396

6.69	300
6.59	317
566.72	463
6.11	284
257.63	279.3
4.91	218
	270
6.98	284
286.02	396
14.32	300
18.13	270
235.33	270
152.67	427
25.79	300
468.31	160.92
10.1	132.98
289	78.97
17.67	
14.94	
25.59	
28.72	216
355.88	355

15.16	218
